# A Novel Hybrid Membrane VAD as First Step Toward Hemocompatible Blood Propulsion

**DOI:** 10.1007/s10439-020-02590-1

**Published:** 2020-09-08

**Authors:** Aldo Ferrari, Costanza Giampietro, Björn Bachmann, Laura Bernardi, Deon Bezuidenhhout, Paolo Ermanni, Raoul Hopf, Sarah Kitz, Gerald Kress, Christian Loosli, Vita Marina, Mirko Meboldt, Giovanni Pellegrini, Dimos Poulikakos, Mathias Rebholz, Marianne Schmid Daners, Tanja Schmidt, Christoph Starck, Georgios Stefopoulos, Simon Sündermann, Bente Thamsen, Peter Zilla, Evgenij Potapov, Volkmar Falk, Edoardo Mazza

**Affiliations:** 1 Laboratory of Thermodynamics in Emerging Technologies, Department of Mechanical and Process Engineering, ETH Zurich, Sonneggstrasse 3, 8092 Zurich, Switzerland; 2 Department of Cardiothoracic and Vascular Surgery, German Heart Institute Berlin, Berlin, Germany; 3 EMPA, Swiss Federal Laboratories for Material Science and Technologies, Überlandstrasse 129, 8600 Dübendorf, Switzerland; 4 Institute for Mechanical Systems, ETH Zurich, 8092 Zurich, Switzerland; 5 Christiaan Barnard Division of Cardiothoracic Surgery, University of Cape Town, Cape Town, South Africa; 6 Laboratory of Composite Materials and Adaptive Structures, ETH Zurich, 8092 Zurich, Switzerland; 7 Product Development Group Zurich, Department of Mechanical and Process Engineering, ETH Zurich, Zurich, Switzerland; 8 Julius Wolff Institute and Center for Musculoskeletal Surgery, Charité – Universitätsmedizin, Berlin, Germany; 9 Laboratory for Animal Model Pathology, Institute of Veterinary Pathology, Vetsuisse Faculty, University of Zurich, Zurich, Switzerland; 10 Department of Cardiovascular Surgery, Charité - Universitätsmedizin Berlin, Berlin, Germany; 11 Deutsches Zentrum für Herz-Kreislaufforschung, Standort Berlin, Germany; 12 Translational Cardiovascular Technologies, Institute of Translational Medicine, Department of Health Sciences and Technology, Swiss Federal Institute of Technology (ETH), Zurich, Switzerland

**Keywords:** VAD, Endothelialization, Wall shear stress, Wall deformation, Hyperelastic membrane

## Abstract

Heart failure is a raising cause of mortality. Heart transplantation and ventricular assist device (VAD) support represent the only available lifelines for end stage disease. In the context of donor organ shortage, the future role of VAD as destination therapy is emerging. Yet, major drawbacks are connected to the long-term implantation of current devices. Poor VAD hemocompatibility exposes the patient to life-threatening events, including haemorrhagic syndromes and thrombosis. Here, we introduce a new concept of artificial support, the Hybrid Membrane VAD, as a first-of-its-kind pump prototype enabling physiological blood propulsion through the cyclic actuation of a hyperelastic membrane, enabling the protection from the thrombogenic interaction between blood and the implant materials. The centre of the luminal membrane surface displays a rationally-developed surface topography interfering with flow to support a living endothelium. The precast cell layer survives to a range of dynamically changing pump actuating conditions i.e., actuation frequency from 1 to 4 Hz, stroke volume from 12 to 30 mL, and support duration up to 313 min, which are tested both *in vitro* and *in vivo*, ensuring the full retention of tissue integrity and connectivity under challenging conditions. In summary, the presented results constitute a proof of principle for the Hybrid Membrane VAD concept and represent the basis for its future development towards clinical validation.

**Electronic supplementary material:**

The online version of this article (10.1007/s10439-020-02590-1) contains supplementary material, which is available to authorized users.

## Introduction

Continuous flow left ventricular assist devices (VADs) represent a routine treatment option for patients in end stage heart failure. In the first 2 years after implantation, the current generation of continuous flow VADs achieves survival comparable to heart transplantation.^21^,^33^ Survival and functional status are markedly improved when compared to medical therapy alone.^38^ On the other side, the generation of supraphysiological and turbulent flow within continuous flow VADs, and the concomitant interaction between blood and the thrombogenic materials at the device’s luminal interface, expose the patients to a life-long risk of thromboembolic events and non-surgical bleeding.^27^,^37^,^39^ At the same time, patients with continuous flow pumps show a pathologic decrease of von Willebrand Factor (vWF) originating from an imbalance between the increased degradation induced by high wall shear stress (WSS) and the decreased endothelial release due to the absence of pulsatility.^5^,^7^,^8^,^31^,^32^,^40^

The preservation of pulsatility represents a target of future device development.^40^ Patients supported with the CARMAT total artificial heart, a pulsatile device partially covered with bovine pericardium at its luminal surface, showed reduced thromboembolic complications without the need for anticoagulation, highlighting the importance of a hemocompatible interface.^17^ The generation of surface textures at the luminal interface of HeartMate XVE supported the formation of a protective biological layer upon patient implantation^12^ and reduced the risk of thromboembolic events without the need of anticoagulation therapy.^35^ However, the overall large pump size, its non-physiological actuation and the uncontrolled generation of a biological layer represent limitations to be addressed.

Full, long-term hemocompatibility can only be bestowed by the combined protection from blood surface interactions inducing thrombosis and from blood damage, caused by non-physiological hemodynamic conditions created by the pump operation.

Here, we address the risk of thrombogenic events triggered by implant materials through the generation of a complete layer of living endothelial cells.^6^,^23^,^34^ In fact, a mature endothelium at the luminal interface of devices that are in contact with blood can provide protection from thrombus formation and subsequent adverse events.^10^

The endothelialization of blood pumps remains however largely unexplored as a result of two independent factors. First, the relative recent surge of pumping devices as destination therapy. In this novel, long-term perspective the interaction between implant materials and blood becomes a matter of concern towards the patient safety and life quality. Second, the non-physiological hemodynamic conditions created by the active implant, as compared to the limited interference created by passive elements such as vascular grafts. In this sense, endothelialization technologies applied to VADs face completely new challenges, which include the formation and/or maintenance of a connected tissue under supraphysiological combinations of WSS and wall deformation (WD).^4^ As most VAD-associated thrombotic events are registered during the first years of support an immediate protection must be considered. For this reason, the *in vitro* generation of a mature endothelial monolayer prior to device implantation must be preferred to ensure the luminal sheltering during the early phases of device actuation.

The actuation of blood pumps yields complex and dynamic gradients of flow-generated WSS which is accompanied by local strain (i.e., WD) when deformable elements are part of the actuation scheme.^14^ In particular, the luminal surface of current VAD features regions of perturbed hemodynamic loads, which prove non-viable for endothelial cells.^1^,^36^ This scenario requires a novel concept to be developed which shall include two converging approaches: (i) The establishment of an alternative pump design, engineered to support a failing heart while minimizing the hemodynamic load and therefore rendering a viable substrate for endothelialization^2^ and, (ii) The implementation of engineered luminal substrates, extending the survival of endothelial cells under highly dynamic and turbulent flow conditions.^4^

Here, we describe the development and validation (*in vitro* and *in vivo*) of a novel pump concept (the Hybrid Membrane VAD; HyMem-VAD) fulfilling the above design criteria. The primary objectives of the presented acute animal study were (i) the development and clinical adaptation of an endothelialization protocol, (ii) the test of safety and actuation performance for the HyMem-VAD, and (iii) the endpoint analysis of cell monolayer integrity upon several hours of pump actuation.

## Materials and Methods

### Membrane Fabrication

**Figure 1:**
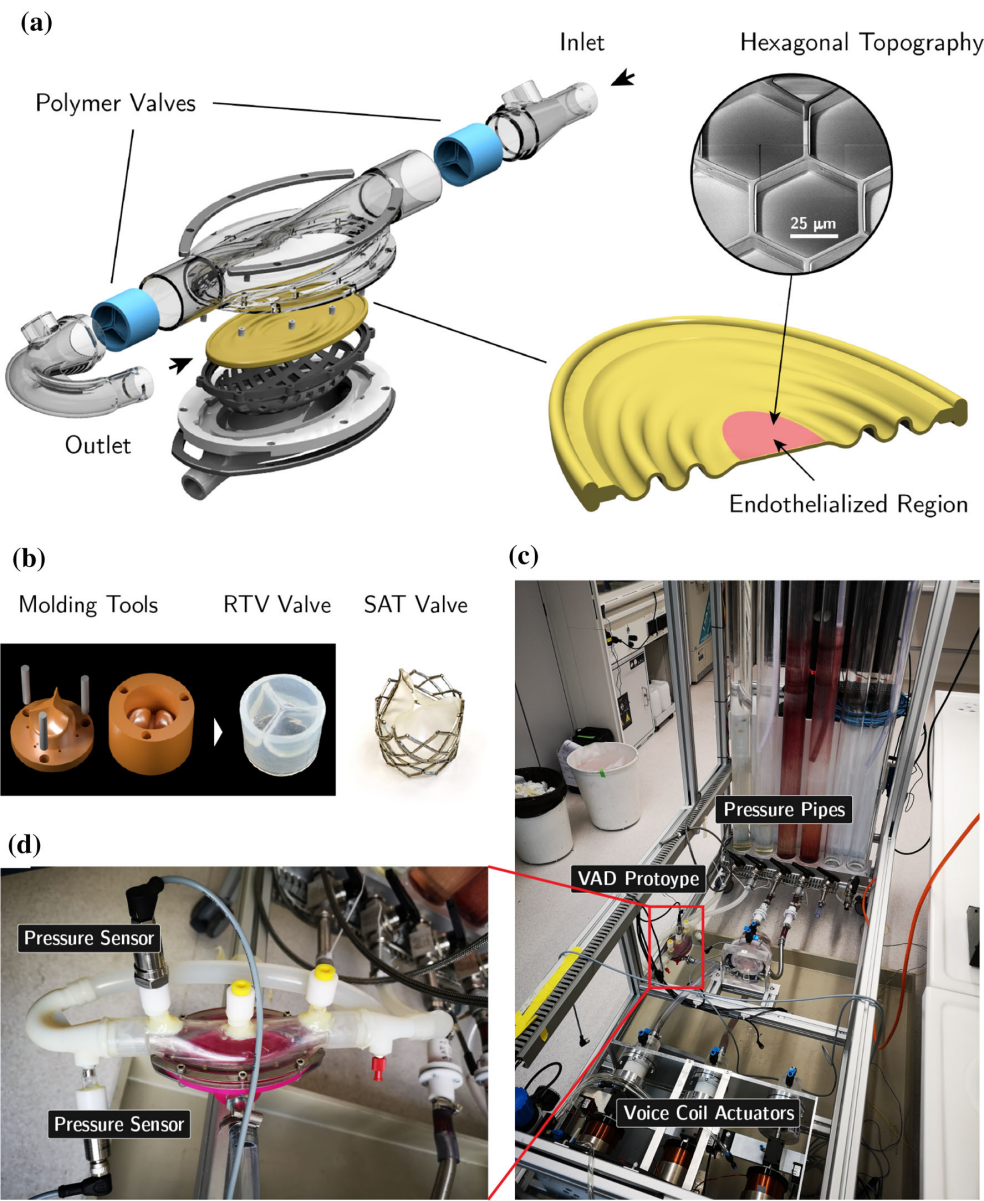
Hybrid Membrane VAD design. (a) The left-hand side shows the full pump assembly, including polymer valves (light blue) and the hybrid membrane (yellow). The right-hand side shows a close up section of the corrugated membrane. Red marks the endothelialized region, which contains the hexagonal surface topography (shown in SEM close-up). Panel (b): The parameterized design of molding tools and the resulting RTV polymer valve are shown on the left-hand side. On the right-hand side a TAVI valve (by SAT) is shown. Panel (c) shows the set-up of the long term test bench. The VAD is connected to vertical pressure cylinders with compliant silicone tubing. A magnification of the pump can be seen in panel (d). This version of the pump is fitted with additional pressure detection locations on the housing, allowing the measurement of pressure gradient across the valves.

The hybrid membrane was generated by molding the hyperelastic RTV 4420 silicone^11^ into an elliptic layer. The chosen design parameters included an aspect ratio of 2:1 and length of the major radius of 44 mm. The membrane features a pattern of corrugations in the cross section protruding along the minor axis (Fig. 1a). The specific design was optimized to reduce the extent of WD generated on the surface upon full membrane extrusion.^19^ In addition, the corrugations are tapered along the major axis to minimize their interference with flow. Upon actuation, the central region of the membrane is cyclically exposed to high levels of WSS (up to 15 Pa) and WD (up to 16% areal strain range), see Refs. 18 and 20. This demanding interface was therefore selected as benchmark to test endothelialization performance. Here, to locally support the adhesion of endothelial cells and the formation and maintenance of a connected endothelial monolayer, honeycomb hexagonal wells with optimized size and geometry (well basal surface = 1045 *μ*m^2^, well height = 6 *μ*m, wall width = 4 *μ*m) were implemented upon membrane fabrication (Fig. 1a^4^). Extended description is provided as supplementary information.

### Pump Design

The HyMem-VAD is a pulsatile, volume displacement pump with design and operation features enabling actuation at high pulsation rates and thus minimizing the overall device volume. The technological core of the pumping scheme is represented by a hyperelastic silicone membrane which is cyclically actuated. The membrane features a pattern of luminal corrugations designed to mitigate the levels of tensile strain upon its full extension. Importantly, the pump design, operation and dedicated topographical patterning^4^ enable luminal endothelialization. We described the most relevant aspects of the *in vitro* pump development.^3^,^11^,^24^,^29^,^36^

Specifically, the pump structure comprises two superimposed chambers horizontally separated by the hybrid membrane (Figs. 1a, 1d). Blood flows in the upper chamber (the blood chamber) while a physiologic saline solution is injected into the lower chamber (actuation chamber). The flow directionality is imposed by one-way valves at the inlet and outlet of the blood chamber (Fig. 1a). In the actuation scheme, a cyclic volume exchange in the actuation chamber induces the controlled extrusion of the hybrid membrane, thus enabling blood propulsion. The hybrid membrane represents therefore the core technological element of this scheme. Extended description of the hybrid membrane fabrication is provided in “Materials and Methods” section.

The pump dimensions were set to yield at a maximum output of 5 l/min while maintaining the apex strain (i.e., at the central region) on the hybrid membrane below 6%. To reduce the necessary stroke volume (SV) and the resulting overall pump size, the operating frequency was raised to 240 bpm (4 Hz^29^). The prototype features in- and outlet polymeric transcatheter valves (Figs. 1a and 1b). Two different valve types were tested: a TAVI valve (Strait Access Technologies, SAT, Cape Town, South Africa) and a custom-developed polymeric tri-leaflet valve (Fig. 1b), made from same silicone elastomer as the propulsion membrane and the coating of the housing (i.e., RTV 4420). The TAVI prosthesis consists of three polyurethane based leaflets, which are connected to a stainless steel stent. The custom made RTV valves were realized based on iterative design and manufactured using a 3D printed negative mold. Finally, the diameter of the chosen cannulae is 20 mm to mitigate WSS by reducing the resulting flow speeds.

### Computational Studies

To evaluate the mechanical and fluid-dynamic loads generated by the pump actuation on the luminal surface of the hybrid membrane, a finite-element and a computational fluid dynamic (CFD) models were developed. The finite-element model rendered the expected membrane deformation, which constituted the input for CFD. This analysis did not consider fluid–structure interactions as for the present hemodynamic conditions no relevant coupling is expected.

**Figure 2:**
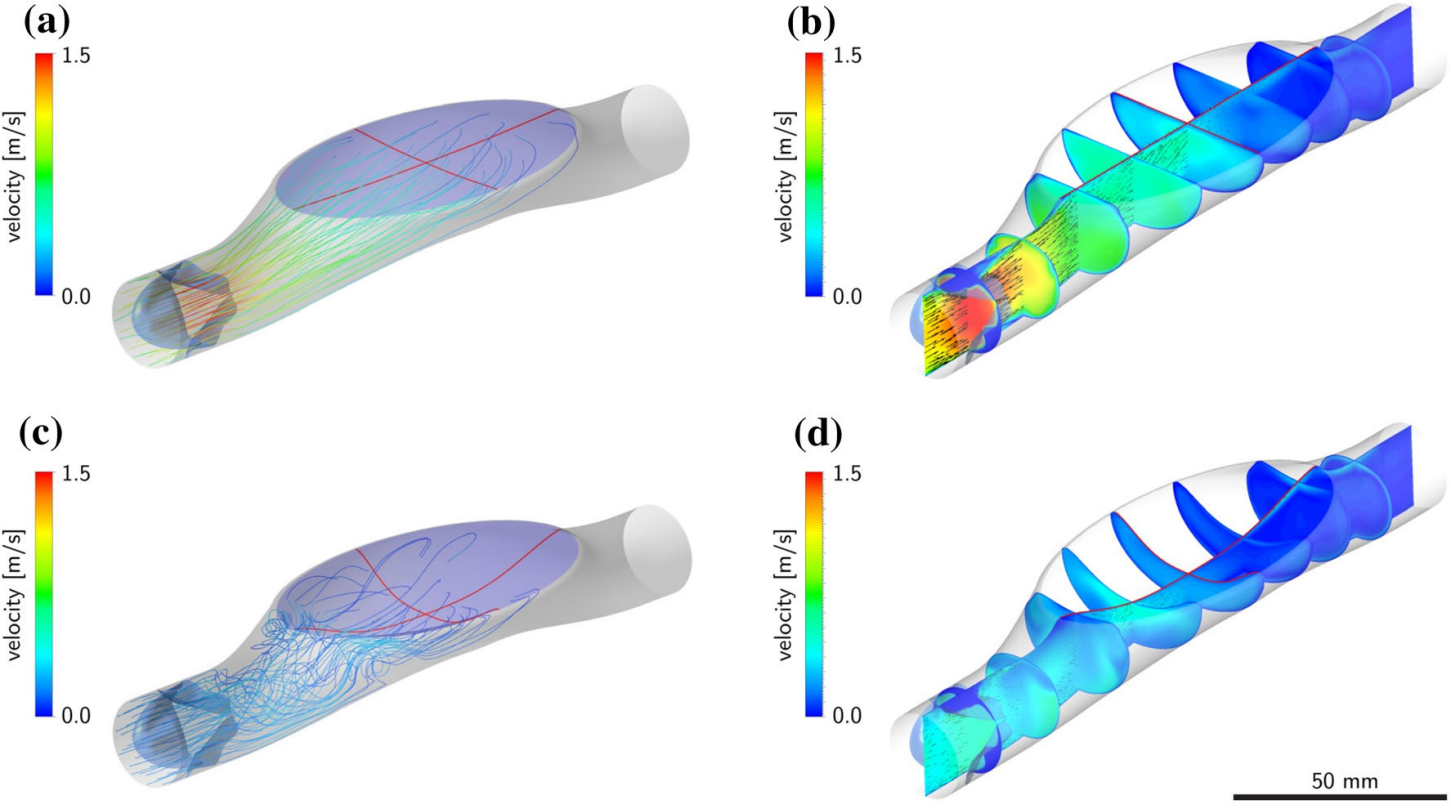
Illustration of the flow field: at the beginning (a and b) and at an intermediate state (c and d) of the diastolic phase. The images show the streamlines (a and c) for selected points at the inlet of the pump obtained from an integration of the corresponding velocity field (b and d).

The flow conditions in the pump were analyzed for different operating conditions in Refs. 18 and 20 and compared with those of a conventional circularly shaped pump chamber indicating improved conditions of WSS for the present elliptical design. Figure 2 shows examples of streamlines obtained from the integration of the corresponding velocity fields during the diastolic phase. For the present study, the analysis of simulation’s results focused on ellipsoidal area with radius of 5 mm, located at the center of the hybrid membrane (Fig. 1).

**Figure 3:**
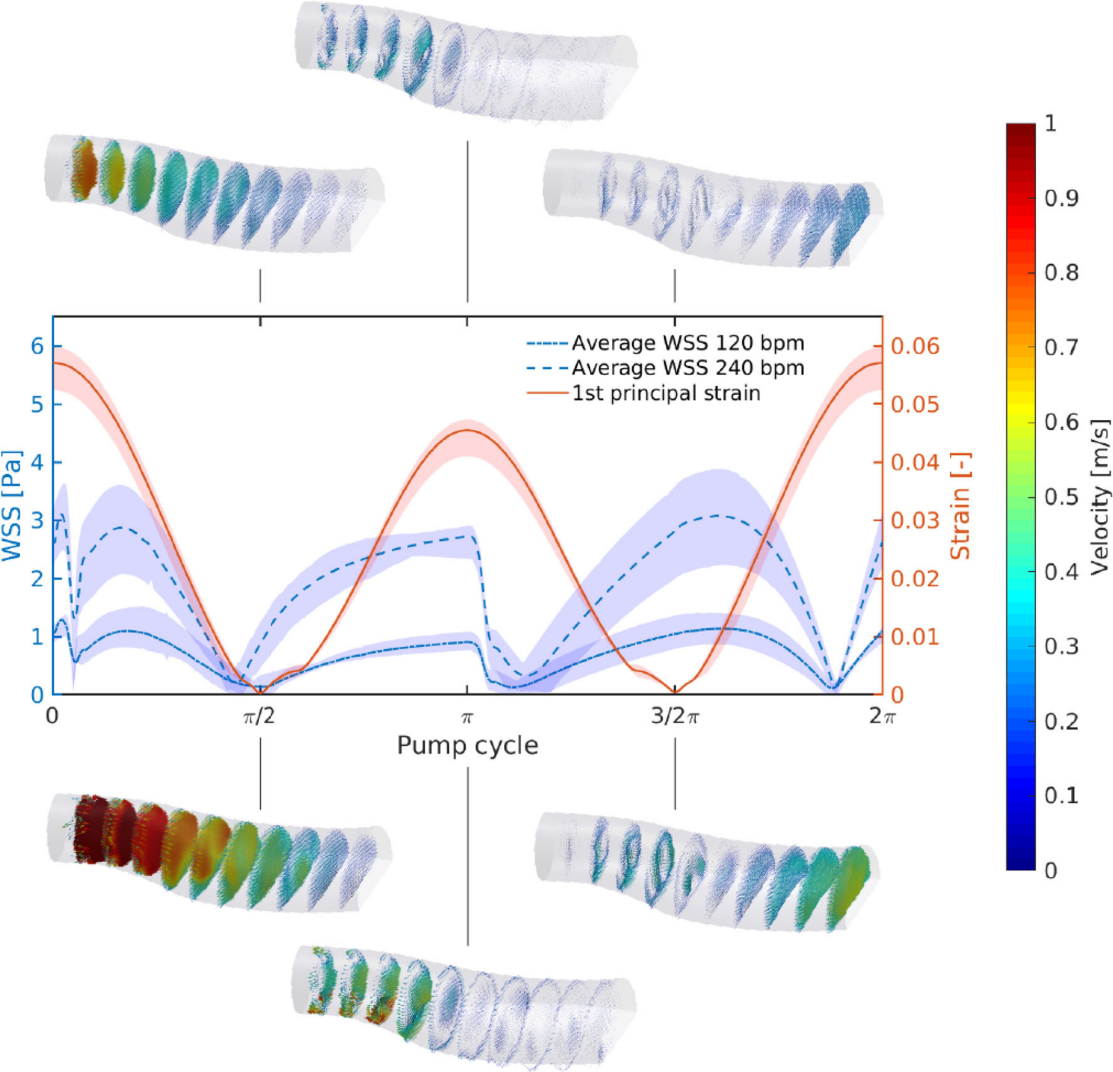
First Principal Elastic Strain and Wall Shear Stress at the cell contacting surface over one pump cycle for a pump frequency of 120 and 240 bpm. Time 0 corresponds to the beginning of the diastole. Sections of the vector-fields from PTV measurements are visualized above (corresponding to 120 bpm) and below (corresponding to 240 bpm). The color-bar corresponding to the measured flow velocity is given on the right-hand side.

The numerical analysis was applied to estimate mechanical strain and fluid stress during pump operation.^20^ Particle tracking velocimetry (PTV) measurements were performed to validate the predicted velocity fields (Fig. 3) as previously reported.^18^^–^20

The WSS at the contacting surface was investigated by performing a non-steady state CFD study of the pump at two operation frequencies of 120 bpm and 240 bpm with a stroke volume of 30 mL.^20^ The results of these calculations, along with those of corresponding finite element simulations of the membrane deformation (see Supplementary Methods) were used to select the portion of the membrane to be covered with endothelial cells. The initial membrane shape was assumed to be flat in order to reduce the complexity of the CFD model. The membrane motion was implemented as a moving boundary into the finite-volume solver ANSYS Fluent 18.2 (ANSYS, “ANSYS Fluent 18.2 User’s Guide.” 2017). Extended description is provided as supplementary information.

### Long-Term *In Vitro* Testing

The long term, cyclic behavior of the hybrid membrane was investigated using a custom-developed long term test bench (Figs. 1c and 1d). This test bench was designed to operate the pump at different actuation frequencies and SV under physiological pressure conditions. Various studies were performed with the whole pump exposed to a range of realistic operating conditions. It could be shown that a long term actuation does not impair the mechanical integrity of the silicone membrane. At 3 L/min pumping rate (realized at 4 Hz actuation frequency and 12.5 mL SV), over 25 million cycles were achieved over a period of 2.5 months. Subsequently, the membrane was retrieved from the pump and visually inspected. No crack initiation sites could be found and also no zones of macroscopic plastic deformation could be identified. The endurance demonstrated in this test is in line with previous investigations on the cyclic behavior of the RTV 4420 silicone used for the hybrid membrane.^11^,^14^ These test results helped defining a safe-range for the actuation settings which were later used in the animal experiments. In order to demonstrate adequate endurance for clinical use, future investigations will involve long term tests with several hundred millions of cycles.

### Cell Culture

**Figure 4:**
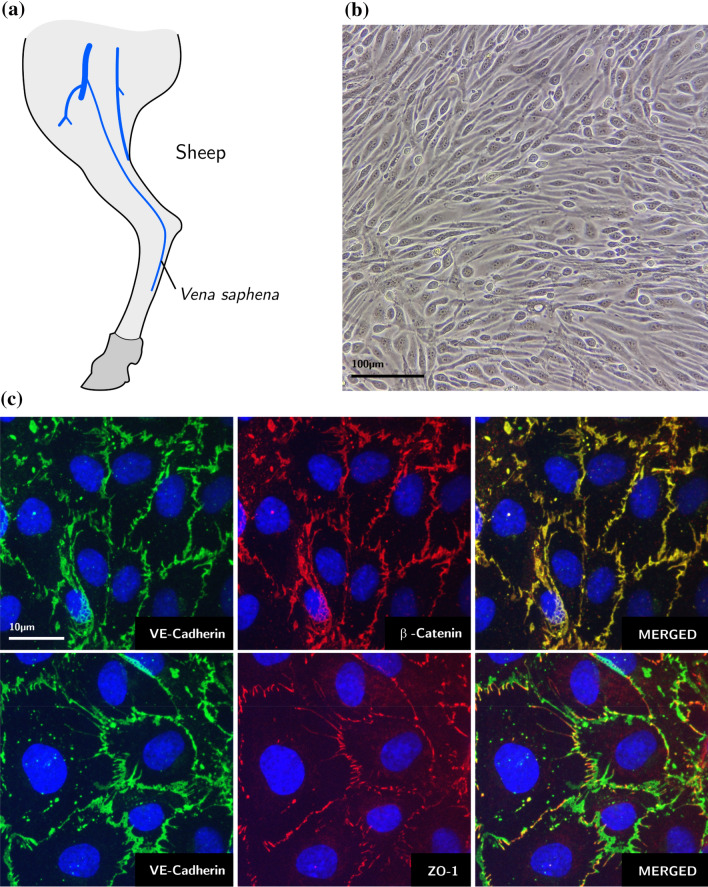
Isolation and characterization of ovine ECs. (a) Representation of the ovine Saphenous vein and (b) bright field image of a typical endothelial monolayer. Scale bar: 100 µm. (c) VE-cadherin, β-catenin and ZO-1 immunostaining of endothelial monolayers. Nuclei are stained with DAPI (blue). Scale bar: 10 µm.

Adult ovine endothelial cells (ECs) were harvested from fresh saphenous vein biopsies (Figs. 4a and 4b). They were grown in medium 200PRF (#M200PRF500, ThermoFisher Scientific) containing LSGS Kit (#S-003-K, ThermoFisher Scientific). The kit includes fetal bovine serum (FBS) 2% v/v; hydrocortisone (1 µg/mL); human epidermal growth factor (10 ng/mL); basic fibroblast growth factor (3 ng/mL) and heparin (10 µg/mL). Cells were maintained at 37 °C and 5% CO_2_. All reported *in vivo* experiments were performed using cells with less than 5 passages *in vitro*. Cells were tested for their ability to reconstitute a mature, growth-arrested endothelial monolayer on a synthetic surface. For this analysis, the target substrate was coated with cross-linked gelatin.^16^ Cells were seeded at high initial density (10^5^ cell/cm^2^).

Tissue generation was assessed after 3 days, through immunofluorescence analysis for endothelial junctional complexes, including VE-cadherin and β-catenin, major components of adherens junctions (AJs^9^), and ZO-1, major component of the tight junctions (TJs^9^). The distribution of cell-to-cell junctions confirmed the generation of fully mature and connected endothelium on target substrates (Fig. 4c). Extended description is provided as supplementary information.

### Antibodies

The following primary antibodies were used: goat polyclonal anti-VE-cadherin (1:200, sc-6458, Santa Cruz Biotechnology Inc.), mouse monoclonal anti-β-catenin (1:100, #610154, BD Biosciences), mouse monoclonal ZO-1 Alexa Fluor 555 (1:100, 1A12, ThermoFisher Scientific). F-actin was stained with TRIC-Phalloidin (SIGMA ALDRICH, P1951). Secondary antibodies were donkey anti-goat–Alexa 488 (A11055, Invitrogen), chicken anti-mouse-Alexa 647 (A21463, Invitrogen).

### Microscopy and Image Processing

Fluorescent images of immunostained samples were acquired with a × 40, 1.3 NA oil immersion objective (Plan Fluor, Nikon, Japan), using a FITC filter, a TRITC filter, a Cy5 filter and a DAPI filter for the cell nuclei. Only adjustment of brightness and contrast were used in the preparation of the figures. For comparison purposes, different sample images of the same antigen were acquired under the same microscopy acquisition settings.

### *In Vivo* Actuation

All experimental protocols were approved by the Landesamt für Gesundheit und Soziales Berlin (LaGeSo, G 205/17), the local German authorities (Number G 0215/17). All methods were carried out in accordance with relevant guidelines and regulations.

The actuation side of the pump housing was connected *via* a hydraulic tube to a PTFE bellows (ElringKlinger AG, Dettingen/Erms, Germany), which in turn was connected to a voice coil (lvcm-095-089-01, Moticont, Van Nuys, CA, USA) that compresses and stretches the bellows. Figure 8 shows a schematic of the actuator. An air bearing (S301301, Newway Air Bearings, Aston, PA, USA) kept the movement of the bellows and voice coil frictionless. As hydraulic fluid a saline solution was used which can be assumed to be incompressible. The stiff hydraulic line additionally allowed neglecting differences in displaced volume between bellows and membrane. The displacement volume of the membrane therefore could be directly correlated to the displacement of the bellows. For the trial the bellows and therefore the membrane were displaced in a sinusoidal way. The actuation parameters, the stroke volume and frequency together with their respective application times are shown in Table 1. By that, the cells were exposed to a broad range of operating points that covers the majority of flow conditions seen in patient application. These experimental conditions were selected for two main purposes: (i) to evaluate endothelial integrity over a wide range of pump operation states and (ii) to verify that the pump can be stably controlled over a wide range of operating conditions. The actuation sequence was selected to create rapid variations of local hemodynamic loads at the luminal surface of the hybrid membrane, therefore challenging the survival of endothelial cells. Extended description is provided as Supplementary Information.

**Table 1:** Overview of pump actuation parameters and their respective application durations

Animal 2			Animal 3			Animal 4		
Frequency (bpm)	Volume (mL)	Duration (min)	Frequency (bpm)	Volume (mL)	Duration (min)	Frequency (bpm)	Volume (mL)	Duration (min)
60	20	2	60	12	0	60	15	1
60	30	31	80	20	1	120	12.5	10
90	20	1	80	30	0	120	15	20
90	30	15	90	30	313	120	20	12
120	30	17				180	15	0
150	20	1				240	12.5	0
160	20	1				240	15	53
180	20	15						
180	30	4						
200	30	6						
240	15	7						

All times were rounded to the next minute. The pump implanted in animal 2 contained no cells

### Sensors

During the trial, the displacement of the membrane was measured by the bellow displacement with a laser sensor (OptoNCDT 1320, Micro-Epsilon Messtechnik GmbH & Co. KG, Ortenburg, Germany) with a sample frequency of 2 kHz. The blood flow through the pump was measured by an ultrasonic flow sensor (SONOFLOW CO.55, SONOTEC Ultraschallsensorik Halle GmbH, Halle, Germany) placed around the outlet graft. Left ventricular, arterial and central venous pressure were recorded through TruWave pressure sensors (Edwards Lifesciences Corp., Irvine, USA). All data was sampled through a MF634 Humusoft Data Acquisition card (Humusoft s.r.o, Prague, Czech Republic) with a sampling frequency of 100 Hz. The data acquisition was running simultaneously to the actuation control on a Real-Time Windows Target environment in MATLAB/Simulink (MathWorks, Inc., Natick, MA, USA).

### Statistical Analysis

**Figure 5:**
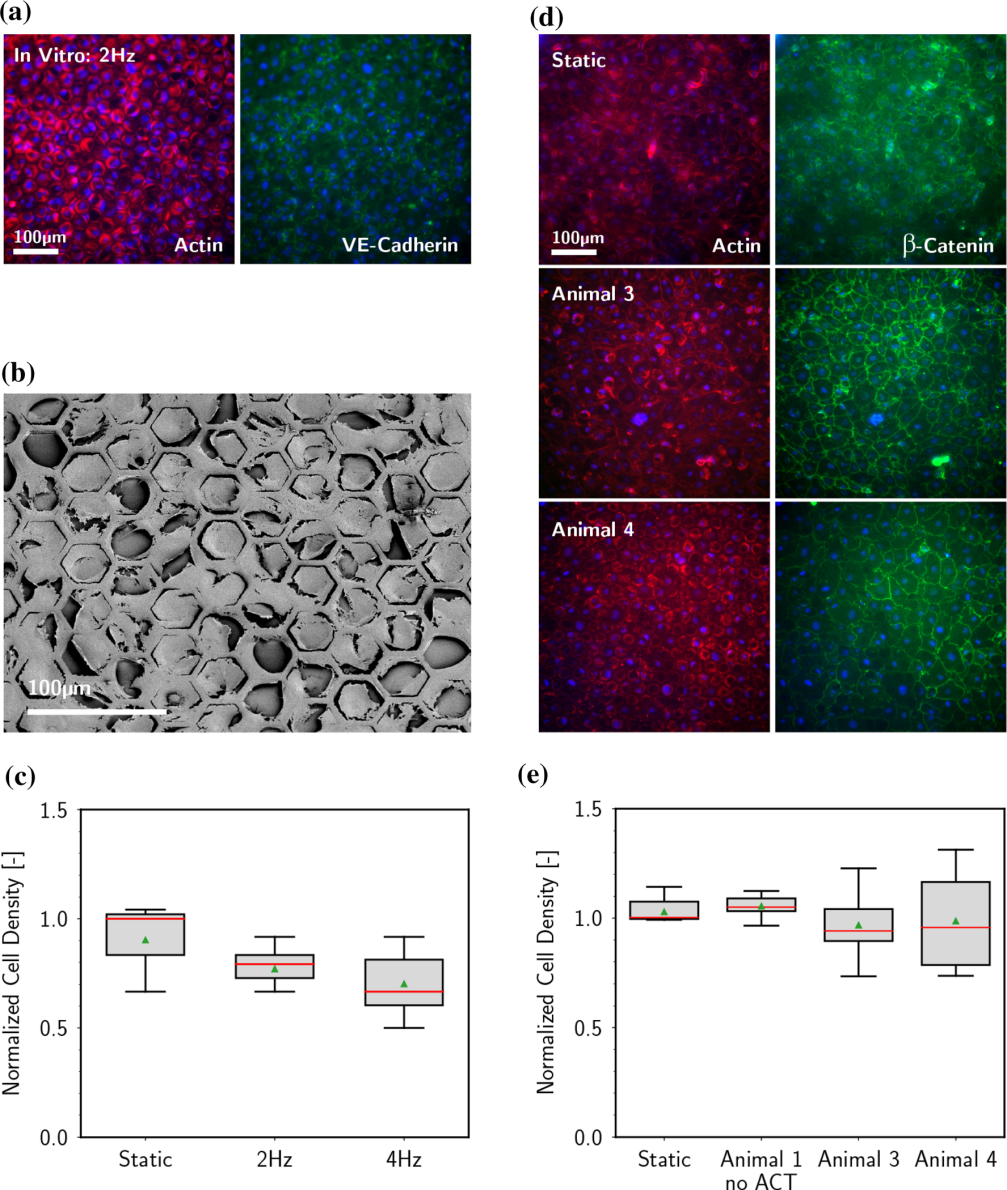
Analysis of endothelialization. *In vitro* test bench: (a) immunofluorescence analysis for actin (red) and VE-cadherin (green). Nuclei are stained with DAPI (blue). Scale bar: 100 µm; (b) SEM analysis of endothelialized hexagonal topography, scale bar: 100 µm, and (c) quantification of the cell density; no statistical significance: 2 Hz vs. static *p* = 0.4, 4 Hz vs. static *p* = 0.2. *In vivo* trials: (d) immunofluorescence analysis for actin (red) and β-catenin (green). Nuclei are stained with DAPI (blue). Scale bar: 100 µm, (e) quantification of the cell density; no statistical significance. Animal 1 vs. static *p* = 0.6, Animal 3 vs. static *p* = 0.4, Animal 4 vs. static *p* = 0.7. Static samples were obtained maintaining the fully mounted device in static conditions in an incubator. These membranes did not undergo the process of implantation. On the other hand, the sample denoted as ‘no actuation’ (Animal 1) was fully implanted but not actuated. In this case the cells interacted with blood and were exposed to flow generated by the animal circulation.

Statistical comparison of cell density was performed using a *t* test. The Shapiro–Wilk test was used to test for normality of data with a significance level of 0.05. The *p*-values reported in Fig. 5 are obtained by comparing several individual measures of cell density obtained in different fields of view for each individual sample. The resulting measure of density variation across the endothelial monolayer is relevant, as the initial phases of denudation (reporting on the failure of the endothelialization strategy and the maintenance of a fully confluent protection) appear with local, restricted loss of cell coverage.

## Results

### *In Vitro* Endothelialization

The *in vitro* test bench was initially exploited to evaluate the performance of the proposed endothelialization approach. The conditions of pump actuation for these preparatory experiments fully reproduced those envisioned for the animal tests.^20^ The central region of the hybrid membrane, featuring honeycomb hexagonal wells (Fig. 1a), was previously coated and seeded with ovine ECs. The selected topographical features support endothelial cell retention without compromising monolayer integrity.^4^ The effect is obtained through a mitigation of the apparent WSS at the apical side of ECs nested in the hexagonal wells, as previously demonstrated by *in vitro* tests under a range of supraphysiological actuating conditions.^4^ In these tests, the topographically-modified surfaces were compared to an equivalent substrate featuring a completely flat surface and demonstrated a superior maintenance of monolayer integrity upon exposure to supraphysiological hemodynamic conditions of combined WSS and WD.^4^

After 3 days of incubation in static conditions, the endothelialized membrane was mounted in the hybrid membrane pump. During the entire procedure the luminal surface was maintained in contact with culture medium and care was taken to avoid bubbles. The prototype was then plugged to the test bench (Fig. 1c) and actuated while the entire device was maintained in an incubated environment. Specifically, the system was subjected to cyclic actuation at 2 or 4 Hz, yielding an average output of 2.5 L/min and 4.0 L/min, respectively. Cell survival and endothelialization performance were assessed through an endpoint immunostaining analysis for junctional proteins and actin cytoskeleton (Fig. 5a). Under all tested conditions, the luminal endothelium remained intact. Endothelial cell-to-cell junctions displayed the typical linear morphology along the boundaries between adjacent cells^22^ and were in contact with cortical F-actin. Importantly, the cell morphology adapted to the underlying hexagonal topography, as previously reported (Fig. 5b^4^). The resulting local cell density was comparable to the control (an identical membrane maintained in static conditions, Fig. 5c). In all, these results demonstrate that the endothelialization protocol and the actuation conditions within the HyMem-VAD (see Materials and Methods) are viable and allow for the generation and maintenance of a connected endothelial monolayer. These achievements represented the prerequisite required to advance the endothelialization protocols to their application in animal trials.

### *In Vivo* Actuation

**Figure 6:**
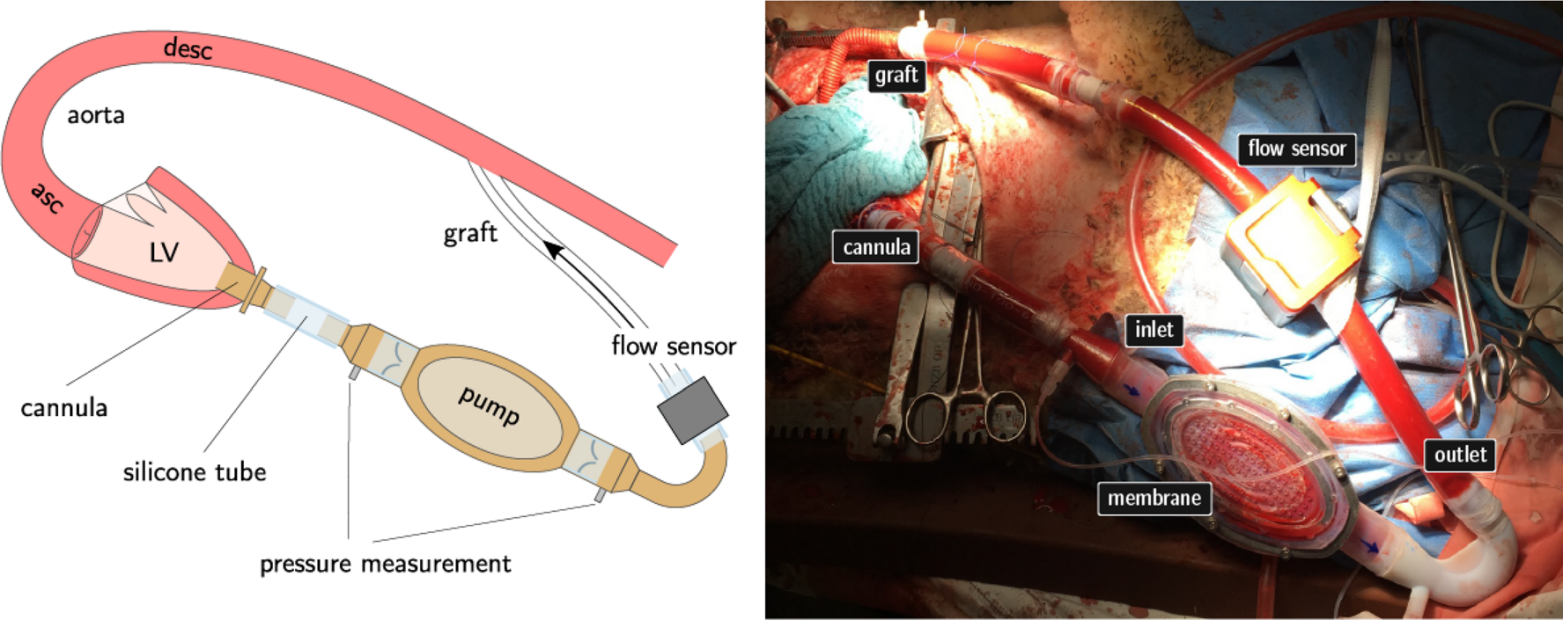
Experimental configuration of *in vivo* trials. Left: schematic representation of the pump configuration including pump housing with polymer valves at in- and outlet position. The inlet of the device is connected to the apex of the left ventricle. The graft on the outlet side connects to the descending aorta. Right: corresponding *in vivo* representation of the set-up during the trial.

**Figure 7:**
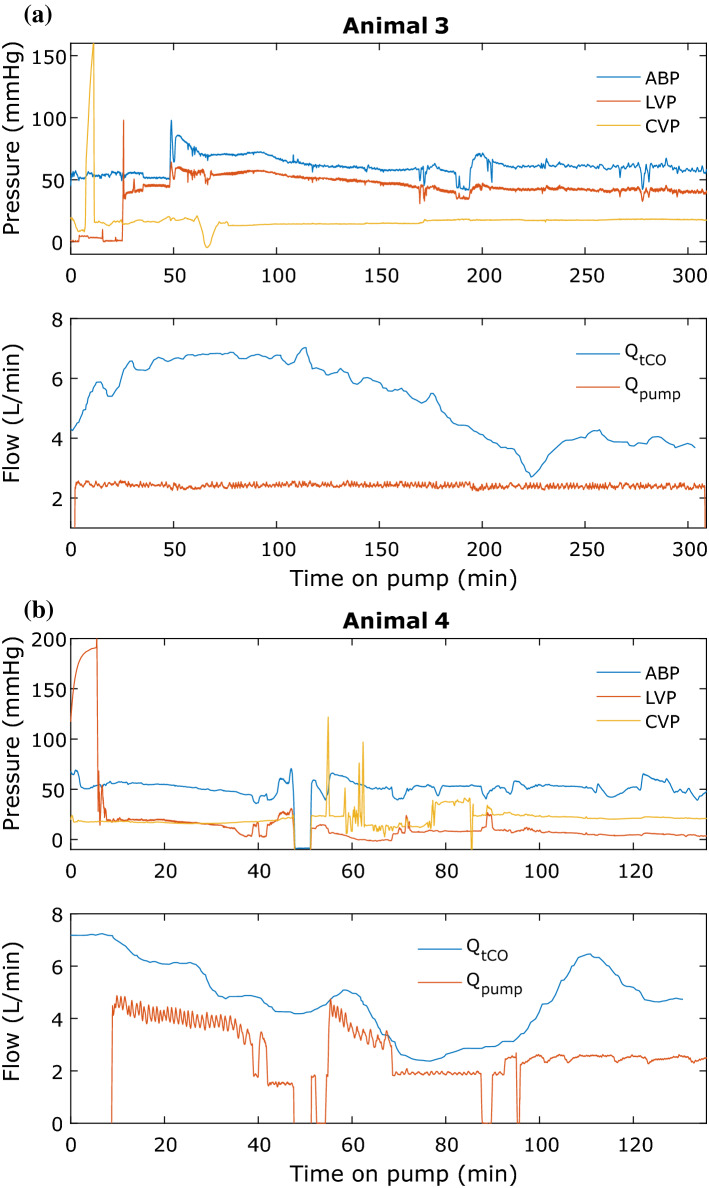
Prevailing pressures and flows during pump operation. The top panels of (a) and (b) show the arterial blood pressure (ABP), left ventricular pressure (LVP) and central venous pressure (CVP) of animal 3 and 4, respectively. The bottom panels of (a) and (b) show the blood flow through the pump (Q_pump_) and total cardiac output (Q_tCO_) consisting of blood flow through the pump and aortic valve combined.

**Figure 8:**
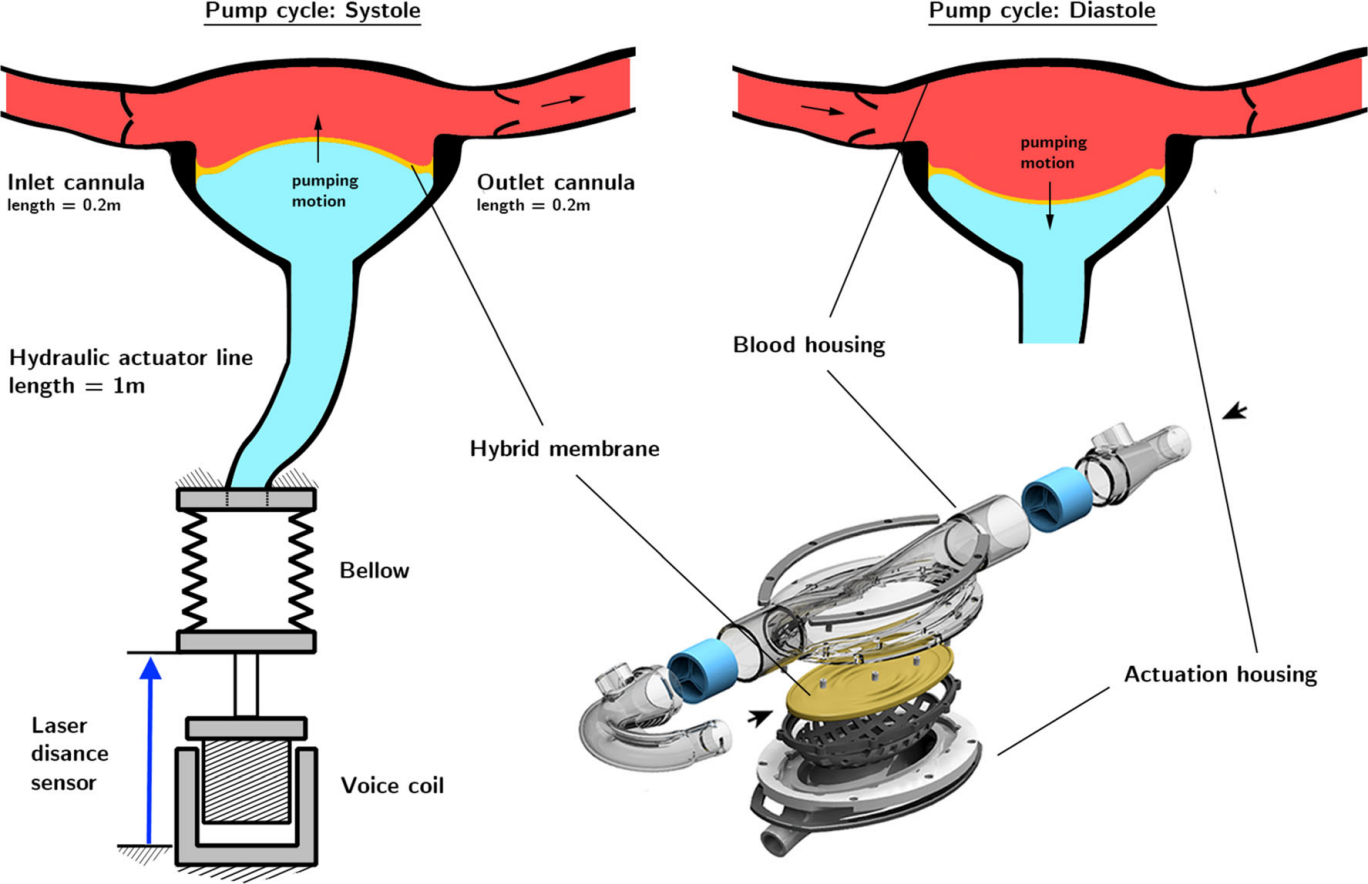
Schematic of pump actuation unit consisting of the HyMem-VAD at the top, a hydraulic line connecting the HyMem-VAD to the bellows, a PTFE bellows and a voice coil that is used to deform the bellows.

All experimental animals (Animal #1–4) were subjected to full implantation of the HyMem-VAD (Fig. 6). A complete description of the surgical settings is provided in Supplementary Materials and Methods. All trials were completed successfully. They allowed for the evaluation of the endothelialization procedure and of different actuation schemes, as previously tested *in vitro* (Figs. 4, 5, 6, 7, and 8).

Specifically, in Animal #1 the pump was endothelialized but not actuated representing therefore a passive element in the circulation. This first test was necessary as internal control for the viability of the endothelialization and mounting procedures, as well as to assess the interaction between cells and blood. Animal #2 served instead as control to minimize the risk of cell damage during the procedure of pump deployment, blood filling, connection with sensors and control system and initial actuation. The second main objective of this experiment was to evaluate the effectiveness of the control system of the pump *in vivo*. To this end, the animal physiological parameters (arterial blood pressure, oxygenation level and heart rate) were monitored for different pump actuation conditions and for the transient states between them. This allowed to verify that the interaction of our pump with the native organ was not causing detrimental effects at the given set-points and during the transition from an operating condition to the next one. The results were used to optimize the experimental procedures and the endothelialization approach in the ensuing animals. In Animals #3 and 4 the HyMem-VAD was endothelialized and actuated, therefore representing the full experimental result of the *in vivo* tests. Complete details on the actuation parameters in these animals are provided in Table 1.

### End-Point Analysis

At the end of each experiment, the HyMem-VAD was cross clamped and carefully removed from the animal body. The actuation lid and the hybrid membrane were dismounted and carefully rinsed with warm medium to clear the surface from blood and preserve the sample. The membranes were then immediately fixed.

To evaluate the performance of the endothelialization procedure and thus the maintenance of a connected endothelial tissue, the fixed specimens were immunostained to reveal the cell nuclei, the actin cytoskeleton and the cell-to-cell junctions (with β-catenin; Fig. 5). All tested experimental configurations (i.e., no actuation, actuation at 2 or 4 Hz) proved viable for ECs. In fact, the retrieved endothelia were completely intact, ensuring a full coverage of the target substrate with no visible discontinuity. The junctional localization of β-catenin confirmed full integrity of the monolayer, which was in all comparable to those obtained in control static conditions (Fig. 5d). Quantitative measurements further showed no significant changes in local cell density upon implant deployment (Fig. 5e).

## Discussion

The presented study demonstrates that the HyMem-VAD implantation protocol and actuation schemes are conducive to device endothelialization, preserving a living endothelium for the entire test duration. Additionally, the endothelialization procedure was successfully adapted to the clinical settings. Altogether, the results demonstrate the feasibility of the innovative concept and represent the necessary basis for its further development. Future validation and translational work will require to secure a complete protection to the implant materials getting in contact with blood. For this to be achieved, we envision a fabrication process that implements the selected topography on the entire luminal surface. The procedure shall be applied to multiple non-planar material interfaces (elastomers, metals, and plastic polymers) therefore requiring optimization of the fabrication protocol. This additional effort will base on the feasibility study presented and will establish the all-round technology for pre-clinical tests, which shall be performed with a fully protected device and tubing. In fact, any bare surface in contact with blood would represent a site for the initiation of thrombotic events, therefore posing a threat to the success of the entire new concept.

### The Hyperelastic Membrane Design and Validation

The hyperelastic membrane represents the technological core of the HyMem-VAD concept (Fig. 1). The design allows a cyclic membrane actuation up to 4 Hz while avoiding the generation of excessive strains at the luminal surface (Figs. 2 and 3). This results in pumping rate of up to 5 L/min and qualifies the HyMem-VAD as potential support for end stage heart failure patients^13^,^15^ and device endothelialization (Fig. 7). The choice of operation at 4 Hz is a limitation of the current design, which however allows the containment of the overall pump dimensions. The aortic pulsatility was not in the scope of this study and no sensor was placed in the vicinity of the aorta. Two separate studies have investigated pulsatile VADs operated at frequencies that exceed the heart rate.^29^,^30^ The *in silico* and *in vitro* results demonstrated that pulsatility remains sufficiently high despite the high frequency of operation. In all, the present work is a unique example of a converging approach in which engineering and biological requirements are considered together during the design phase. It demonstrates that physiologically-relevant actuation and device endothelialization are possible and compatible in a heart failure support device (Fig. 8).

The current pump design and overall size are optimized towards its intended experimental use and not to achieve implantability. The prototype features a large flange, clamping rings and screw connections to clamp the two halves of the pump housing to facilitate mounting and demounting of membranes and valves. Additionally, the cannulas are designed for extracorporeal animal experiments and specifically to visually assess the state of the pump. For this purpose the outlet cannula features a big curvature as shown in Fig. 1.

Future development for clinical translation will consider a more compact design. In particular, for a clinically-optimized prototype such design elements (flange, clamping rings and screw connections) will be eliminated and the residual volume conformed by the actuation. The blood chamber with connections for the cannulas will be similarly reduced in size. In parallel, we envision a new design of the cannulas and pump body to achieve an implantation configuration with curved inlet cannula and straight outlet. The reduction of the pump size through the incorporation of increased stroke frequencies is instrumental in this approach.

### The *In Vitro* Endothelialization Approach

The endothelialization of artificial substrates has been applied to attain *in vivo* protection of vascular grafts.^6^ In this case, successful coverage was based on the *in vitro* generation of a precast autologous endothelium.^41^ Alternatively, porous materials have been applied to foster efficient transmural ingrowth.^26^ More recently, attempts to extend this procedure to vascular stents exploited the *in vivo* regenerative potential of circulating endothelial precursors, locally recruited to the luminal interface.^25^,^28^ VAD endothelialization involves tissue generation and maintenance under complex flow conditions^36^ which can be locally mitigated by the implementation of surface topographies interfering with flow.^4^

The here-reported tests demonstrate that a rationally-designed honeycomb topography (Fig. 1) can support the generation and maintenance of a fully-connected and functional endothelium (Figs. 4 and 5) in representative regions of the HyMem-VAD featuring a dynamically changing hemodynamic landscape.^18^,^20^ The same fabrication approach can be extended to other regions of the system. In addition, the precast endothelium was generated using cells isolated from the *Vena Saphena*, which represents a potential source of endothelialization for cardiovascular devices in human patients. Based on these considerations, the endothelialization protocol can theoretically be upscaled to cover the entire luminal surface of the HyMem-VAD.

From the biological point of view, we have obtained a sound proof that the chosen materials, the selected interface topography, the endothelialization protocols, the implantation procedure, and the actuation *in vivo* are all compatible with the survival of a reconstituted endothelium. Endothelial cells and tissues are very delicate and would immediately suffer from non-viable conditions. Therefore, the maintenance of tissue integrity after several hours of actuation is a non-trivial technical challenge and undoubtedly provides a proof of viability on which we can leverage for our future developments. Altogether, these results demonstrate that the HyMem-VAD is compatible with device endothelialization based on the *in vitro* generation of a precast endothelium. Alternative endothelialization mechanisms, including the contribution of circulating endothelial precursors, may play an additional supportive role.

The response of the endothelium, and of the entire cardiovascular system, upon exposure to high frequency pulsations for extended periods of time will be addressed by the future development of our novel concept. This investigation shall be pursued by means of advanced *in vitro* bioreactors, enabling the long-term actuation of the HyMem-VAD, and chronic animal studies. Altogether, these future efforts shall provide a clear evaluation of the proposed trade-off between low WSS and high actuation frequency.

### Limitations

The present study has several limitations. All reported results were obtained in acute *in vivo* tests during which the animals were constantly under full anesthesia. Therefore, our conclusions are restricted to the validation of the clinical procedure for pump endothelialization, assembly, implantation and initial actuation. Further chronic experiments will be necessary to evaluate the survival of the endothelium and its long-term adaptation to the pump function *in vivo*. In addition, the extended time necessary for the preparation and implantation of the pump system contributed to define, and limit, the duration of the actuation phase. The limited time window during which the experimental animals showed stable physiological parameters defined the duration of the actual experiments. The luminal endothelialization of the HyMem-VAD was restricted to a central portion of the hyperelastic hybrid membrane. This was selected due to the prevailing values of hemodynamic stresses to which cells are exposed during pump actuation. While 5 L/min are considered a relevant level of support for clinical application, extension to 8–10 L/min flow represent a desirable feature for a wider applicability of the heart pump. Correspondingly, our future experiments will investigate the feasibility of device actuation and cell viability under increased level of flow.

## Electronic supplementary material

Below is the link to the electronic supplementary material.

Supplementary file1 (DOCX 34 kb)
