# Supplementary material for: A Novel Hybrid Membrane VAD as First Step Toward Hemocompatible Blood Propulsion
Source: Ann Biomed Eng. 2020 Sep 8;49(2):716–31. doi: 10.1007/s10439-020-02590-1 (PMC7851026; doi:10.1007/s10439-020-02590-1)
Supplement: Supplementary file 1 — Supplementary file1 (DOCX 34 kb) [file 10439_2020_2590_MOESM1_ESM.docx]

**Supplementary Materials and Methods**

***Membrane Fabrication (Extended)***

The mold for the corrugated membrane consists of two parts (a top and a bottom), between which the silicone elastomer is cured. Mold components were milled from Polyoxymethylene (POM) using a high accuracy CNC milling machine. The mold was equipped with two small outlet holes, which allow excess silicone to flow out during the molding process. Further, in all four corners of the mold, centring pins are positioned in corresponding holes. This allows the controlled closing of the mold during manufacturing. The fabrication of the elastomer membrane encompasses four main steps. The first step is preparing the liquid silicone, followed by pouring it into the mold, then closing the mold inside a vacuum chamber and curing the silicone. Finally, the membrane is removed from the mold.

The silicone preparation starts by mixing both components of the room temperature vulcanizing (RTV) silicone elastomer by weight the appropriate mass ratio provided by the manufacturer (in this case 1:1). The elastomer used in this application is Silbione**®** RTV 4420 (Elkem, Oslo, Norway). This material cures within two hours at room temperature and has a Young’s modulus of E = 455 MPa (2) and the Poisson’s ratio of v = 0.5 (corresponding to an incompressible material). After curing, the RTV 4420 bonds only weakly to the POM the mold, and therefore is very easy to detach and remove from the mold without further use of any mold release agent. After both components are thoroughly mixed by hand for 5 min, the resulting viscose silicone has a working time of 30 min. During the first 10 min of this time, the silicone was always de-gassed in a vacuum chamber in order to remove all the air inclusions which have been introduced during the mixing process. Before pouring the silicone into the mold, the latter was cleaned with ethanol and compressed air was used to get rid of all remaining dust particles. A dedicated device was developed that then allows the closing of the mold inside the vacuum chamber. This prevents the formation of air inclusions in the membrane.

***Generation of Hexagonal Topography***

The template wafers featuring the hexagonal honeycomb arrays were produced by standard lithography process, as previously reported (1).

***Computational Studies (Extended)***

The unstructured mixed grid with 1.6 x 10^6^ elements was developed in ICEM CFD with the global size of the tetrahedron elements set to 1.37 mm. Ten prism layers with a height growth rate of 1.15 and an initial height of 0.06 mm were used to resolve the boundary layer. The dynamic mesh method, (ANSYS, “ANSYS Fluent 18.2 User’s Guide.” 2017) including smoothing and remeshing, was used to allow large grid deformation. The grid quality was checked through a mesh sensitivity study for one specific pump configuration (7) to minimize computational costs and limiting spatial discretization errors.

The density of the fluid was set to *ρ = 1050 kg/m^3^* and the shear-strain rate dependent dynamic viscosity was modelled with the Carreau model (3; 4). The volume flow at the outlet is increasing up to 22 l/min and the resulting Reynolds number is expected to reach a value up to 6000. Therefore, the turbulence was modeled using the k-ω-SST-model given by Menter (8). The solver accuracy was set to double precision and all residual targets for continuity, momentum and turbulence are set to a value of 10^-5^. Each pump cycle consists of 3200 time steps and 4 cycles were calculated to achieve a time periodic convergence.

A non-linear finite element simulation was performed to analyze the membrane deformation behavior and the resulting luminal strain. The problem symmetry allowed restricting the model to a quarter of the membrane geometry. Realistic material properties (2) were implemented in the simulation. A positive and negative homogeneous pressure were then applied at the actuating side of the membrane and the magnitude was increased until the desired total SV (i.e. 30 ml) with physiologic pressures was achieved.

The flow conditions in the pump were analyzed for different operating conditions in (6) and compared with those of a conventional circularly shaped pump chamber indicating improved conditions of WSS for the present elliptical design. Figure 2 shows examples of streamlines obtained from the integration of the corresponding velocity fields during the diastolic phase. For the present study, the analysis of simulation’s results focused on ellipsoidal area with radius of 5 mm, located at the center of the hybrid membrane (Fig. 1). The first principal strain and WSS along the actuation cycle are reported in Fig. 3. The principal elastic strain $(\varepsilon_{1}$) increases up to 6% during the phase of positive inflation and to 4.8% during the opposite negative phase. Note that the second principal strain is also positive and displays values that are about half those of the maximum principal strain, while the third one is negative, due to the out of plane contraction of the membrane. The WSS remained low for the entire actuation cycle. The expected maximum WSS for the actuation at 2 Hz is 1.5 Pa. This value increased to 3.9 Pa when the cycle frequency doubled (4 Hz).

***Cell Isolation, Culture and Immunostaining (Extended)***

Pieces of ovine saphenous veins (about 3 cm length), collected at the Veterinary Hospital of the University of Zurich, were rinsed in Hank's Balanced Salt Solution (HBSS, #14025092, ThermoFisher Scientific) containing 2.5 µg/ml of Amphotericin B (#A2942, SIGMA ALDRICH), 100 U/ml of Penicillin and 100 mg/ml of Streptomycin (Penicillin-Streptomycin, # 15140122, SIGMA ALDRICH). The edges of the vessel were then clamped, filled with a solution of 0.5 mg/ml Collagenase A (#10103578001, Roche) in HBSS and incubated at 37°C for 20 min. The solution containing cells were then collected, centrifuged and the cells were re-suspended in medium 200PRF (#M200PRF500, ThermoFisher Scientific) containing LSGS Kit (#S-003-K, ThermoFisher Scientific. The kit includes fetal bovine serum (FBS) 2% v/v; hydrocortisone (1 µg/ml); human epidermal growth factor (10 ng/ml); basic fibroblast growth factor (3 ng/ml) and heparin (10 µg/ml)). The cell suspension was placed in a 9 cm^2^ well plate and the cells were grown until confluence (about 10 days).

A majority of ECs comprised the resulting cell population (Fig. 4B) which however featured a small (~2%) contamination from other vascular cells, including smooth muscle cells, pericytes, and myofibroblasts. Optimal purity (~100%) was obtained after a step of FACS-based purification. Confluent monolayers were trypsinized, centrifuged and the pellet was re-suspend in a solution of PBS containing 1% BSA and 2mM EDTA to avoid clump formation. The cell suspension was then incubated for 40 min on ice with 1 µg/ml of anti-Platelet/endothelial cell adhesion molecule (Pecam1)-FITC conjugated antibody (#ABIN182060, antibodies-online GmbH). After the incubation cells were centrifuged and the pellet was re-suspended in PBS containing 1% BSA and 2mM EDTA and the cells were FACS sorted. The positive cells were collected in PBS containing 2% FBS (the FBS used was part of the iLSGS Kit, #S-003-K, ThermoFisher Scientific) and put back in culture. After 1 week of expansion in culture, cells were immunostained for VE-cadherin in order to verify the purity of the cell population. In general, the harvesting process yielded a primary population of ~2x10^6^ ECs per cm of vein biopsy.

The coating and seeding protocols were optimized for the *in vitro* and *in vivo* assessment of tissue survival upon actuation of the HyMem-VAD. This adaptation required the development of a magnetic stencil to confine cell seeding to the target central region (Fig. 1) and to support cell growth during incubation. Specifically, the membranes were cleaned with 70% ethanol and rinsed three times with PBS before starting the coating procedure. They were subsequently coated with 1,5% gelatin (104070, Merck Millipore, USA) (9) and stored at 4°C until the seeding of the cells. Importantly, the gelatin coating procedure applied to the substrates does not alter surface topography (1).

At the end of the endothelialization experiments, cells were fixed for 20 min with 4% paraformaldehyde (PFA) at room temperature. The cells were next permeabilized with 0,5 % Triton x-100 in PBS for 10 min. After washing the samples three times for 5 min with PBS, they were incubated in 5% w/v bovine serum albumin (BSA; Sigma-Aldrich, USA) in PBS for 2 h at room temperature. The samples were incubated with the primary antibodies (See Antibodies section) overnight at 4^o^C. Subsequently, the samples were rinsed 2 times for 10 min with 5% BSA in PBS. They were then incubated with the corresponding secondary antibodies for 45 min at room temperature. Finally, the samples were washed 4 times for 30 min with PBS. For staining of nuclei, Hoechst was added at 10 μg ml^-1^ during a washing step.

***Pump preparation***

For the trial the custom designed pump housing (Fig. 1; (5)) was connected with ventricular-aortic cannulation (Fig. 6). For the connection procedure the pump was assembled and de-aired, filling the blood chamber containing the seeded cells with cell culture media. In a subsequent step the blood chamber was sealed by clamping the inlet and outlet cannula, with the membrane in its center position. The pump remained in the incubator and was brought to the operation theater just prior to implantation. Before unclamping the seal the cannulation was thoroughly checked for any remaining air bubbles and the actuation side was filled with saline solution and the fluid level adjusted such that the center position of the actuator corresponded with the center position of the membrane.

***In vivo actuation (Extended)***

Animal #3 was supported for a period of 313 min during which the mean central venous pressure varied between 13 mmHg and 20 mmHg. The arterial pressure averaged between 57 mmHg and 90 mmHg with the pulse pressure, calculated as $PP=\max\left( P_{ar} \right)-min(P_{ar})$, ranging between 20 mmHg and 50 mmHg. The blood flow through the pump (Q_pump) remained constant at 2.4 L/min while the total cardiac output (Q_tCO) dropped from 6.1 L/min to 2.7 L/min and then recovered to 3.8 L/min. Figure 7 shows the prevailing pressures and flows during pump operation in this experiment.

Animal #4 was supported with higher stroke frequencies and lower SV. Figure 7 displays the prevailing pressures and flows during pump operation. In particular, for the first 39 minutes the animal was supported with 240 bpm and a SV of 15 mL. Over this time the resulting pump flow decreased from 4.4 L/min to 3.5 L/min while the mean LVP and CVP remained constant between 15 and 18 mmHg. The mean arterial blood pressure dropped slightly from 48 to 42 mmHg as the Q_tCO reduced from 7.1 to 4.8 L/min. After this period the actuation was reduced to 120 bpm. After briefly stopping and restarting the pump actuation, 55 min into the trial, all pressures and flows were back to previous levels (Fig. 7).

***Animal Preparation and Anesthesia***

All animals underwent a standard check upon arrival, after which they have 1 additional week for acclimatization and adaptation. The day before the operation in early afternoon the animals were fed for the last time according to the standard diet. Water was accessible to the animals at all times until the anesthesia. In the stall sedation was done by means of intramuscular injection of 0.6 mg/kg of Midazolam. The peripheral venous catheter was installed for intravenous access through ear- and peripheral leg veins. A bolus of Trapanal was injected for a deeper sedation with initial concentration 10-15 mg/animal, adjusted if necessary. The animal was intubated orotracheally and transferred to the operation room under bag valve mask where it was connected to mechanical ventilation. The total intravenous anesthesia was maintained with Midazolam 0.2-0.4 mg/kg/h combined with Fentanyl 5-15 μg/kg/h under mechanical ventilation. A feeding tube was inserted for extraction of gastric acid and prevention of inflation. The bladder catheterization was performed for a better balance of liquids and anesthesia management. The oropharyngeal temperature sensor was inserted. The probe for transesophageal echocardiogram was inserted. The operating surfaces, the point for arterial line and other relevant locations on the animal were shaved, cleaned and disinfected.

During the operation preparation the vital parameters were controlled. The volume was managed with electrolytic solution and hydroxyethyl starch (HAES). During administration of surgical anesthesia the parameters were controlled with a clinical hemodynamic monitoring.

***Animal Trial Surgery***

All experimental protocols were approved by the Landesamt für Gesundheit und Soziales Berlin (LaGeSo, G 205/17), the local German authorities (Number G 0215/17). All methods were carried out in accordance with relevant guidelines and regulations. Animals were under general anesthesia. Orotracheal intubation was applied for respiration. Antiarrhythmic drugs were given before the procedure and maintained with continuous perfusion to prevent ventricular fibrillation. Animals were placed on the right side with the left thoracic side in a slightly supine position. A left sided thoracotomy was performed to enter the thoracic cavity.

After administering heparin, the pericardium was opened by a longitudinal incision to reach the apex. A towel was placed below the heart to expose the apex. The suture ring was placed at the ideal spot at the apex, which was identified by manual compression under echocardiographic visualization. The margins of the ring were marked with a pen at the surface of the heart as reference for the suture line. 2-0 Prolene sutures were placed in a circular fashion at this marking with felt-pledged u-sutures. The sutures were then placed in the Gore-Tex-part of the apex-ring and tightly knotted. Afterwards the apical whole was produced with a cutter, producing an apical whole to house the apex cannula.

The apical cannula was previously connected to a tube, which was occluded with a tube clamp and later on was used to connect the pump. As a next step the descending aorta was identified and the adventitial tissue was partly removed to have save access to the vessel. The descending aorta was clamped with a partial clamp. After incision of the vessel wall, a 10F Dacron craft was sutured end-to-side. Before removal of the partial clamp, the graft was occluded with a tube clamp. The graft was connected to a tube which was later on used to connect the system (Fig. 6). 30 min before finalization of the surgical procedure, the pump was prepared to avoid delay. After finalization of the preparation of the pump it was immediately connected to the apical cannula and the graft at the descending graft. The clamps were disconnected and blood could flood the primed chamber. The pump was set at function immediately after declamping.

**Supplementary References**

1. Bachmann BJ, Giampietro C, Bayram A, Stefopoulos G, Michos C, et al. 2018. Honeycomb-structured metasurfaces for the adaptive nesting of endothelial cells under hemodynamic loads. *Biomater Sci*

2. Bernardi L, Hopf R, Ferrari A, Ehret AE, Mazza E. 2017. On the large strain deformation behavior of silicone-based elastomers for biomedical applications. *Polym Test* 58:189-98

3. Doost SN, Zhong L, Su B, Morsi YS. 2016. The numerical analysis of non-Newtonian blood flow in human patient-specific left ventricle. *Comput Methods Programs Biomed* 127:232-47

4. Karimi S, Dabagh M, Vasava P, Dadvar M, Dabir B, Jalali P. 2014. Effect of rheological models on the hemodynamics within human aorta: CFD study on CT image-based geometry. *J Non-Newton Fluid* 207:42-52

5. Loosli C, Moy L, Kress G, Mazza E, Ermanni P. 2018. Corrugated diaphragm shape design study for hemocompatible pulsatile ventricular assist devices. *Comput Methods Biomech Biomed Engin*:1-9

6. Loosli C, Rupp S, Thamsen B, Rebholz M, Kress G, et al. 2019. High-frequency operation of pulsatile ventricular assist devices: A computational study on circular and elliptically shaped pumps. *Int J Artif Organs*:391398819857442

7. Loosli C. 2020. *Numerical and experimental studies for the development of an endothelialized ventricular assist device*. ETH

8. Menter FR. 1994. Two-equation eddy-viscosity turbulence models for engineering applications. *AIAA Journal* 32:1598-605

9. Stefopoulos G, Giampietro C, Falk V, Poulikakos D, Ferrari A. 2017. Facile endothelium protection from TNF-alpha inflammatory insult with surface topography. *Biomaterials* 138:131-41
